# Characterization of Dendritic Cells and Myeloid-Derived Suppressor Cells Expressing Major Histocompatibility Complex Class II in Secondary Lymphoid Organs in Systemic Lupus Erythematosus-Prone Mice

**DOI:** 10.3390/ijms252413604

**Published:** 2024-12-19

**Authors:** Felipe R. Uribe, Fabián González-Martínez, Sebastián A. Echeverría-Araya, Alison Sepúlveda-Pontigo, Karissa Chávez-Villacreses, Andrés Díaz-Bozo, Isabel Méndez-Pérez, Valentina P. I. González, Karen Bohmwald, Alexis M. Kalergis, Jorge A. Soto

**Affiliations:** 1 Millennium Institute on Immunology and Immunotherapy, Laboratorio de Inmunología Traslacional, Departamento de Ciencias Biológicas, Facultad de Ciencias de la Vida, Universidad Andrés Bello, Santiago 8370133, Chile; f.uribeleiva@uandresbello.edu (F.R.U.); f.gonzlezmartnez@uandresbello.edu (F.G.-M.); s.echeverraaraya@uandresbello.edu (S.A.E.-A.); a.seplvedapontigo@uandresbello.edu (A.S.-P.); k.chavezvillacreses@uandresbello.edu (K.C.-V.); a.dazbozo@uandresbello.edu (A.D.-B.); i.mndezprez@uandresbello.edu (I.M.-P.); v.gonzalezhenriquez@uandresbello.edu (V.P.I.G.); 2 Instituto de Ciencias Biomédicas, Facultad de Ciencias de la Salud, Universidad Autónoma de Chile, Santiago 8900000, Chile; karen.bohmwald@uautonoma.cl; 3 Millennium Institute on Immunology and Immunotherapy, Facultad de Ciencias Biológicas, Pontificia Universidad Católica de Chile, Santiago 8330025, Chile; akalergis@bio.puc.cl; 4 Departamento de Endocrinología, Facultad de Medicina, Pontificia Universidad Católica de Chile, Santiago 8320000, Chile

**Keywords:** systemic lupus erythematosus (SLE), MRL/MpJ-Fas^lpr^/J, innate immunity, myeloid-derived suppressor cells (MDSCs), dendritic cells (DCs), major histocompatibility complex class II (MHC-II), co-stimulatory molecules

## Abstract

Systemic lupus erythematosus (SLE) is an autoimmune disease characterized by self-antibody production and widespread inflammation affecting various body tissues. This disease is driven by the breakdown of immune tolerance, which promotes the activation of autoreactive B and T cells. A key feature of SLE is dysregulation in antigen presentation, where antigen-presenting cells (APCs) play a central role in perpetuating immune responses. Dendritic cells (DCs) are highly specialized for antigen presentation among APCs. At the same time, myeloid-derived suppressor cells (MDSCs) can also express MHC-II molecules, although their role in SLE is less understood. Utilizing the SLE model, MRL/MpJ-*Fas^lpr^*/J, we determined the presence of different phenotypes of DCs and MDSCs expressing MHC-II in secondary lymphoid organs, along with the gene expression of *ICOSL*, *CD80* and *CD86* in the spleen. Our study determined that the most abundant population of APCs in secondary lymphoid organs corresponds to cDC CD103^−^CD11b^+^ MHC-II^+^ throughout SLE development. Additionally, *ICOSL* expression increased over time, becoming more preponderant in week 16 in the SLE model, which could indicate that it is a crucial pathway for the development and progression of the pathology. In week 16, we observed a positive correlation between M-MDSC MHC-II and IFN-γ-producing CD4^+^ T cells.

## 1. Introduction

Systemic lupus erythematosus (SLE) is a chronic autoimmune disease that can potentially affect various body tissues, resulting in heterogeneous clinical manifestations among subjects [1]. Worldwide, the incidence of SLE varies between 1.5–11 persons per 100,000 inhabitants [2]. Within this, women are more often affected than men [1,2]. SLE has no defined etiology since genetic and environmental factors promote its development and progression [3,4]. One of the key factors for SLE development is the loss of immune tolerance, which induces the production of autoreactive cells and self-antibodies [3]. This triggers a chronic inflammatory state produced by the innate and adaptive immune system [4].

The innate immune system primarily comprises myeloid cells, most of which can present antigens, known as antigen-presenting cells (APCs) [5,6]. Professional APCs present antigens to CD4^+^ T cells through the major histocompatibility complex class II (MHC-II) [7]. APCs in an SLE context are hyperactivated, hence an increase in autoantigen presentation [8]. T cell activation requires not only the interaction between MHC and T cell receptor (TCR) but also the presence of co-stimulatory molecules [9]. The co-stimulatory molecules significantly impacting SLE include SLAMF6, ICOSL, CD80, CD86, CD40, and OX40L [9].

Dendritic cells (DCs) are considered APCs par excellence [10] since these cells can migrate to secondary lymphoid organs, such as the spleen and lymph nodes, and present antigens to T cells [11]. In these tissues, DCs present antigens to naive T cells, triggering T cell activation and polarization, which subsequently migrate to peripheral tissues [11]. DCs have different phenotypes, including conventional DCs (cDCs), plasmacytoid DCs (pDCs), and monocyte-derived DCs (moDCs), among others [12]. In patients and murine models with SLE, DCs are altered in abundance [13]. SLE patients especially show decreased cDCs, while increased pDCs are observed [14,15]. This phenotype of DCs promotes SLE development through the secretion of type I interferon (IFN-I) [16]. Notably, this cytokine impacts the DCs themselves, promoting activation by CD80, CD86, and MHC-II, promoting T cell activation [13].

Other myeloid lineage cells, which have become research subjects in chronic diseases such as cancer and autoimmunity, are myeloid-derived suppressor cells (MDSCs) [17]. In murine models, MDSCs present a monocytic (M-MDSCs) (CD11b^+^Ly6C^high^Ly6G^−^) and granulocytic (G-MDSCs) (CD11b^+^Ly6C^−/low^Ly6G^+^) phenotype [18]. MDSCs are characterized by their immunosuppressive activity [18]. However, in SLE, these cells do not have a defined role since some authors attribute anti-inflammatory characteristics to these cells, while others propose that they promote inflammation [19,20,21]. In cancer, it has been observed that MDSCs express MHC-II [22,23]. The expression of co-stimulatory molecules in MDSCs, including CD40, CD80, and CD86, has also been determined [22,24,25]. In autoimmunity, specifically in experimental autoimmune encephalomyelitis (EAE), it has been described that M-MDSCs express MHC-II together with CD80 and CD86 [26].

As antigen presentation via MHC-II is hyperactivated in SLE, we evaluated phenotypes associated with antigen presentation by DCs and MDSCs throughout the pathology. We hypothesize that distinct populations of DCs and MDSCs expressing MHC-II show an increase during SLE development in secondary lymphoid organs. Therefore, we aim to characterize different phenotypes of DCs and MDSCs expressing MHC-II in spleen and axillary lymph nodes throughout the pathology using MRL/MpJ-*Fas^lpr^*/J strain, an acute murine SLE model. Additionally, we evaluated the transcriptional levels of co-stimulatory molecules, specifically *ICOSL*, *CD80*, and *CD86*, in the spleen at various points of the SLE. This is in conjunction with the correlation presented by MDSCs expressing MHC-II concerning CD4^+^ T cells. Our results indicate that the secondary lymphoid organs of the MRL/MpJ-*Fas^lpr^*/J model show an increase in specific phenotypes of DCs and MDSCs. We mainly observed a significant presence of cDC CD103^−^CD11b^+^ MHC-II^+^. Moreover, we observed that *ICOSL* expression in our SLE murine model remains increased over time. This may indicate that it promotes the activation and polarization of Th_2_ and Th_17_ cells, enhancing the inflammatory environment. Moreover, we observed that in the spleen of the SLE model, there exists a positive correlation between M-MDSC MHC-II with IFN-γ-producing CD4^+^ T cells at week 16, which could indicate that this phenotype of MDSCs could be promoting the pathology.

## 2. Results

### 2.1. The MRL/MpJ-Fas^lpr^/J Model Shows an Increase in Clinical Score, Proteinuria, and Anti-dsDNA Antibodies Throughout the SLE

**Figure 1:**
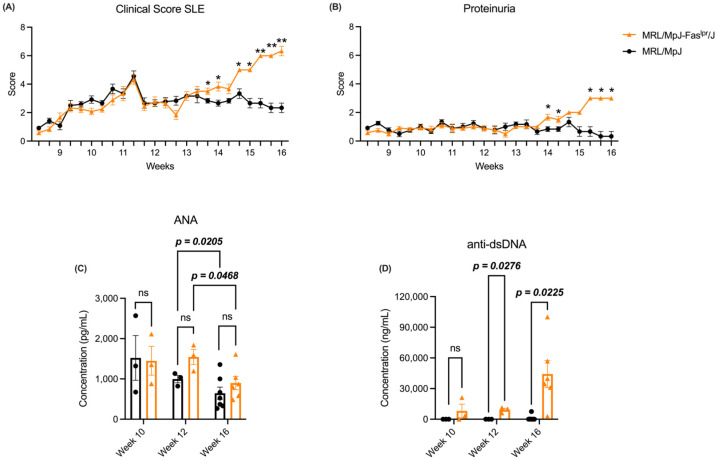
The MRL/MpJ-Fas^lpr^/J model did not present variations in ANA levels. However, clinical score, proteinuria, and anti-dsDNA antibody concentrations increased throughout the study. Physical and biochemical parameters were evaluated to determine the clinical score, proteinuria, and self-antibody concentration. (**A**) Clinical score and (**B**) proteinuria were measured throughout the SLE development. MRL/MpJ (black circles) and MRL/MpJ-Fas^lpr^/J (orange triangles). The statistical analysis utilized was a two-way ANOVA post-Tukey’s test, * *p* < 0.05 and ** *p* < 0.01. (**C**) ANA and (**D**) anti-dsDNA antibody concentration from week 10 to week 16. MRL/MpJ (black circles) and MRL/MpJ-Fas^lpr^/J (orange triangle). MRL/MpJ week 10 (*n* = 3), MRL/MpJ-Fas^lpr^/J week 10 (*n* = 3), MRL/MpJ week 12 (*n* = 3), MRL/MpJ-Fas^lpr^/J week 12 (*n* = 3), MRL/MpJ week 16 (*n* = 7), MRL/MpJ-Fas^lpr^/J week 16 (*n* = 6). The statistical analysis used was a two-way ANOVA post-Tukey’s test, *p* < 0.05 (bold), “ns” indicated not significant.

The evaluation of clinical parameters is key to ascertaining that our murine model presents an SLE-like condition, within which biochemical and physical parameters were evaluated (Table S1), as well as the concentration of antinuclear antibodies (ANA) and anti-dsDNA. Since week 14, we observed an increase in the clinical score of the MRL/MpJ-*Fas^lpr^*/J model (Figure 1A). The same effect is reflected in proteinuria, since an increase in this parameter was observed from this point of the study in the SLE group (Figure 1B). On the other hand, ANA showed a similar pattern between both experimental groups regardless of the week (Figure 1C). However, a decrease in the concentration of this autoantibody was observed from week 12 onwards in both groups (Figure 1C). Regarding anti-dsDNA antibodies, these showed an increase in the SLE group, specifically at weeks 12 and 16 (Figure 1D). These data suggest that the MRL/MpJ-*Fas^lpr^*/J murine model develops an SLE-like phenotype as the week progresses.

### 2.2. The Spleen of MRL/MpJ-Fas^lpr^/J Mice Shows an Increase in DCs and M-MDSCs Compared to the Axillary Lymph Node at Week 16

**Figure 2:**
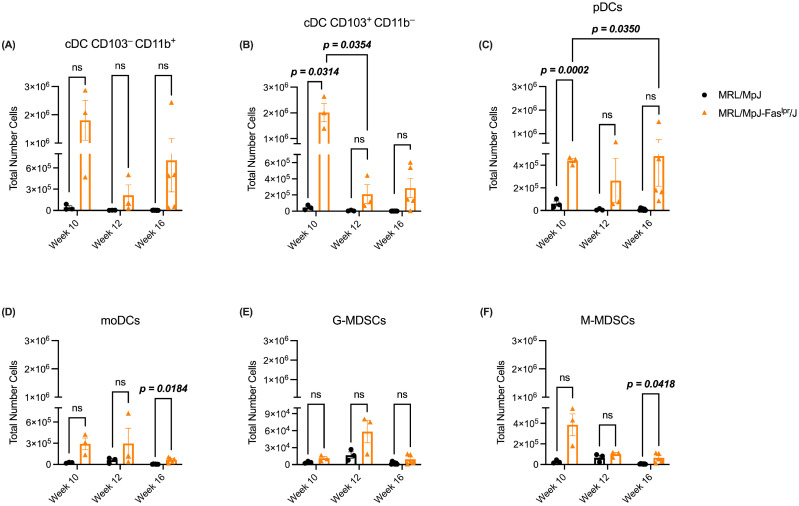
cDC CD103^+^CD11b^−^ and pDCs increased in axillary lymph nodes in the SLE model at week 10, whereas moDCs and M-MDSCs increased later in our study. The abundance of DCs and MDSCs throughout the pathology was analyzed from the axillary lymph nodes of both murine models. A total number of (**A**) cDC CD103^−^CD11b^+^, (**B**) cDC CD103^+^CD11b^−^, (**C**) pDCs, (**D**) moDCs, (**E**) G-MDSCs, and (**F**) M-MDSCs in the different weeks of the study. MRL/MpJ (black circles) and MRL/MpJ-Fas^lpr^/J (orange triangle). MRL/MpJ week 10 (*n* = 3), MRL/MpJ-Fas^lpr^/J week 10 (*n* = 3), MRL/MpJ week 12 (*n* = 3), MRL/MpJ-Fas^lpr^/J week 12 (*n* = 3), MRL/MpJ week 16 (*n* = 7), MRL/MpJ-Fas^lpr^/J week 16 (*n* = 5). The statistical analysis used was a two-way ANOVA, post Tukey’s test. *p* < 0.05 (bold), “ns” indicated not significant.

Since myeloid lineage cells significantly impact SLE development, we characterized the presence of DCs and MDSCs in secondary lymphoid organs throughout SLE development. Firstly, we evaluated different populations of DCs and MDSCs in axillary lymph nodes. According to this, cDC CD103^−^CD11b^+^ and G-MDSCs in this tissue did not show variations over time or between experimental groups (Figure 2A and Figure 2E, respectively). On the other hand, both cDC CD103^+^CD11b^−^ and pDCs presented an increase in week 10 for the SLE group (Figure 2B and Figure 2C, respectively). However, cDC CD103^+^ CD11b^−^ decreased at week 12, a trend which was maintained until week 16 (Figure 2B). Meanwhile, pDCs increased from week 10 to week 16 (Figure 2C). Regarding moDCs and M-MDSCs, both cell types increased in number, specifically in week 16, according to the SLE model (Figure 2D and Figure 2F, respectively).

**Figure 3:**
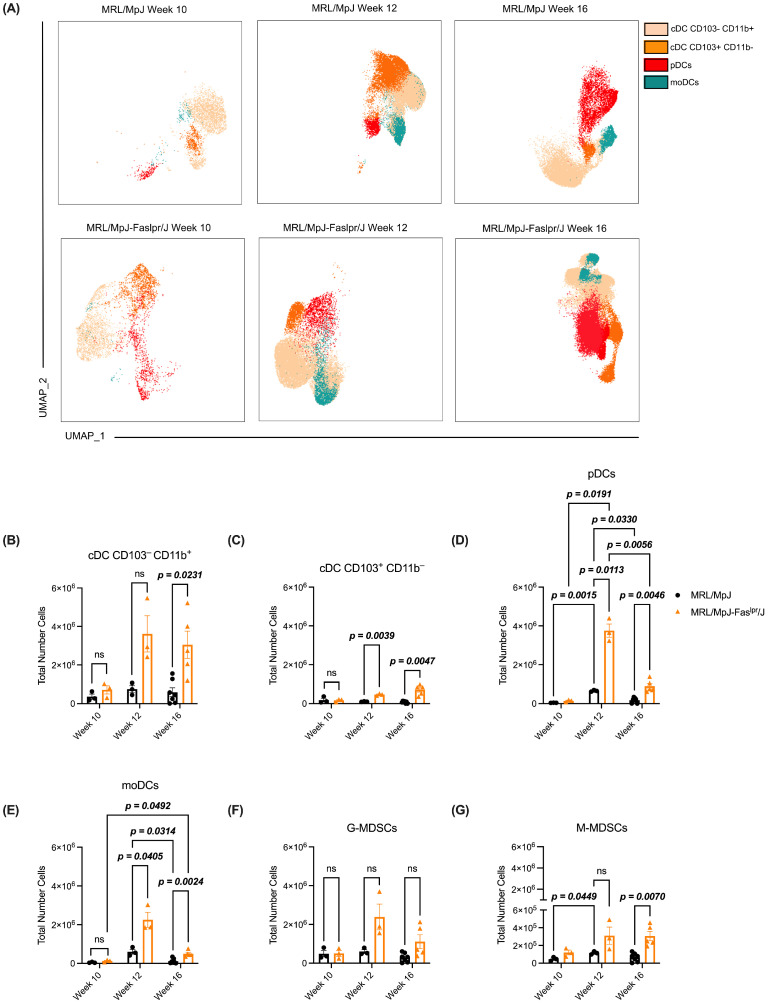
The different phenotypes of DCs increased between weeks 12 and 16 in the spleen of the SLE model. Meanwhile, M-MDSCs presented this behavior in week 16. The abundance of DCs and MDSCs throughout the pathology was analyzed from the spleens of both murine models. (**A**) UMAP represents the behavior of cDC CD103^−^CD11b^+^ (beige), cDC CD103^+^CD11b^−^ (orange), pDCs (red), and moDCs (green) in the clusters throughout the different weeks in both experimental groups. A total number of (**B**) cDC CD103^−^CD11b^+^, (**C**) cDC CD103^+^CD11b^−^, (**D**) pDCs, (**E**) moDCs, (**F**) G-MDSCs, and (**G**) M-MDSCs at the different points of the study (weeks 10, 12, and 16). MRL/MpJ (black circles) and MRL/MpJ-Fas^lpr^/J (orange triangle). MRL/MpJ week 10 (*n* = 3), MRL/MpJ-Fas^lpr^/J week 10 (*n* = 3), MRL/MpJ week 12 (*n* = 3), MRL/MpJ-Fas^lpr^/J week 12 (*n* = 3), MRL/MpJ week 16 (*n* = 7), MRL/MpJ-Fas^lpr^/J week 16 (*n* = 5). The statistical analysis used was a two-way ANOVA post-Tukey’s test, *p* < 0.05 (bold), “ns” indicated not significant.

On the other hand, these cells were evaluated in the spleen to understand further the populations of DCs and MDSCs in secondary lymphoid organs. We performed a dimensional reduction analysis (UMAP) in the spleen, in which we observed the distribution of DCs of interest (Figure 3A). In week 10, we observed that both experimental groups presented less dense clusters (Figure 3A). Meanwhile, from week 12, the clusters begin to be more defined and robust (Figure 3A), consistent with the increase in the number of cells observed in both weeks (Figure 3B–E). Additionally, we observed at weeks 12 and 16 in both experimental groups’ specific populations of DCs that share sections of the clusters (Figure 3A), especially cDC CD103^−^CD11b^+^ cells with moDCs (Figure 3A). Also, we analyzed the number of DCs and MDSCs in the spleen (Figure 3B–G). Within this, we observed that cDCs CD103^−^CD11b^+^ present an increase at week 16 in the SLE phenotype (Figure 3B). Meanwhile, cDCs CD103^+^CD11b^−^ increased concerning the control from week 12 (Figure 3C). On the other hand, pDCs presented a variable behavior throughout the weeks, showing a peak at week 12 in both experimental groups (Figure 3D). However, the SLE group showed a higher abundance of these cells (Figure 3D). Meanwhile, moDCs showed an expansion from week 10 to week 16 in SLE mice; this increase is seen at weeks 12 and 16 (Figure 3E). Additionally, we analyzed the two phenotypes of MDSCs, whose G-MDSC profile did not show any variation across weeks (Figure 3F). Nevertheless, the M-MDSC phenotype in the SLE group increased in week 16 (Figure 3G). Furthermore, we analyzed the immunosuppressive molecules characteristic of MDSCs (Arg-1 and iNOS) (Figure S1A–D). Both markers are expressed in practically all MDSCs independently of the experimental group (Figure S1A–D). These results suggest that the SLE model’s axillary lymph nodes and spleen exhibit different behaviors concerning the number of DCs and MDSCs. Especially in the spleen of the MRL/MpJ-*Fas^lpr^*/J model during week 16, an increase of cDC CD103^−^CD11b^+^, cDC CD103^+^CD11b^−^, pDCs, moDCs, and M-MDSCs is highlighted. Their immune populations could have an impact on the development and progression of the pathology.

### 2.3. cDCs CD103^−^CD11b^+^ Expressing MHC-II Represent the Majority Cell Population Within the DCs and MDSCs Analyzed in the Spleen of the MRL/MpJ-Fas^lpr^/J Model

**Figure 4:**
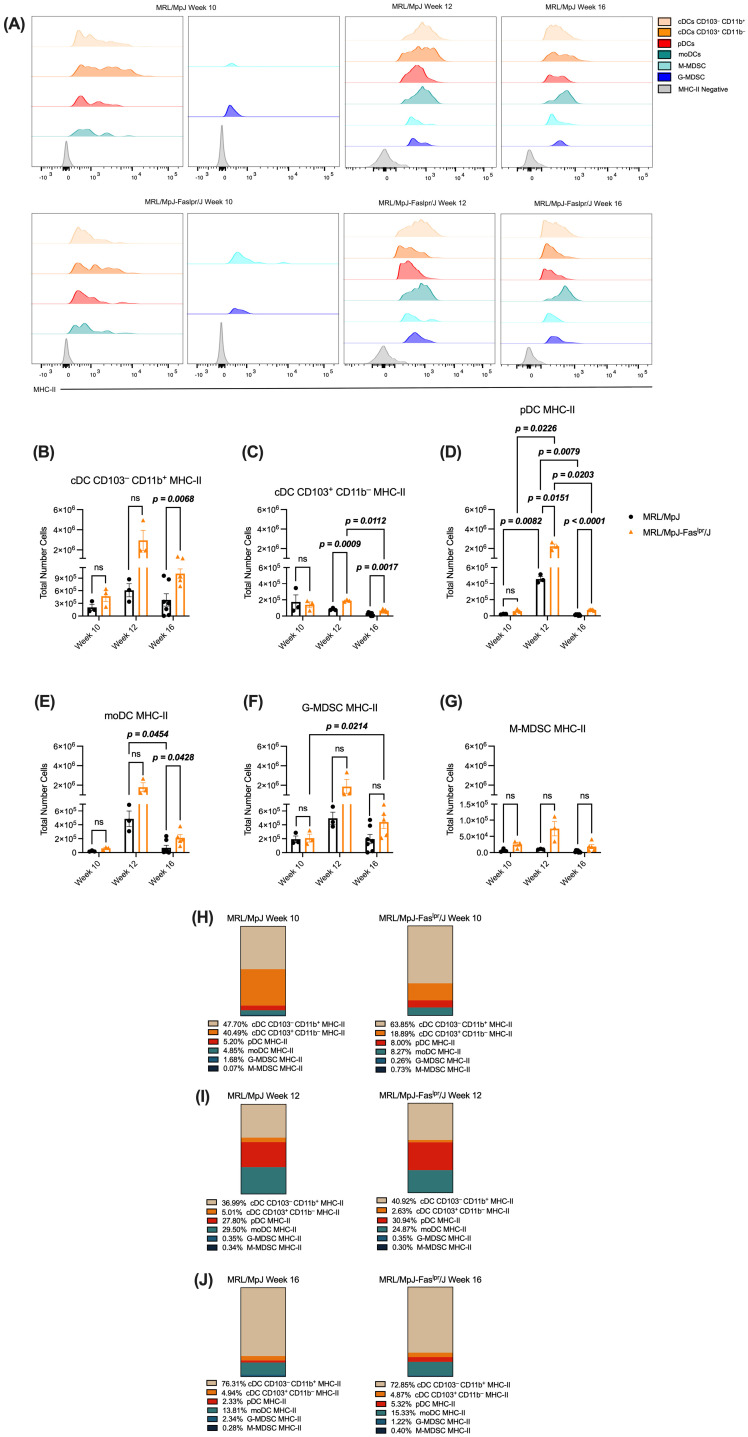
The number of cDC CD103^−^CD11b^+^ MHC-II^+^ during week 16 increased in SLE and was positioned as the most representative population across weeks in the MRL/MpJ-Fas^lpr^/J model. The abundance of DCs and MDSCs expressing MHC-II throughout the pathology was analyzed from the spleens of both murine models. (**A**) Histograms corresponding to MHC-II in the different weeks in each of the immune populations analyzed. MHC-II negative (gray), cDC CD103^−^CD11b^+^ (beige), cDC CD103^+^CD11b^−^ (orange), pDCs (red), moDCs (green), G-MDSCs (blue), and M-MDSCs (sky blue). The histogram for week 10 is divided into a left panel based on the BV605 fluorochrome and a right panel based on the PerCP-Cy5.5 fluorochrome. The histograms for the weeks are also based on the PerCP-Cy5.5 fluorochrome. A total number of (**B**) cDC CD103^−^CD11b^+^, (**C**) cDC CD103^+^CD11b^−^, (**D**) pDCs, (**E**) moDCs, (**F**) G-MDSCs, and (**G**) M-MDSCs expressing MHC-II at the different points of the study. MRL/MpJ (black circles) and MRL/MpJ-Fas^lpr^/J (orange triangle). MRL/MpJ week 10 (*n* = 3), MRL/MpJ-Fas^lpr^/J week 10 (*n* = 3), MRL/MpJ week 12 (*n* = 3), MRL/MpJ-Fas^lpr^/J week 12 (*n* = 3), MRL/MpJ week 16 (*n* = 7), MRL/MpJ-Fas^lpr^/J week 16 (*n* = 5). The statistical analysis used was a two-way ANOVA post-Tukey’s test, *p* < 0.05 (bold), “ns” indicated not significant. (**H**–**J**) Population abundance of DCs and MDSCs expressing MHC-II in the spleen during weeks 10, 12, and 16, respectively.

To understand the abundance of potential APCs in the spleen, we analyzed the expression of MHC-II in the cell populations that had been previously characterized. We observed that each immune population of interest expressed MHC-II at different weeks and independently of the experimental group (Figure 4A). Regarding the number of cells, both cDCs CD103^−^CD11b^+^ MHC-II and moDCs MHC-II presented an increase during week 16 in the SLE group (Figure 4B and Figure 4E, respectively). On the other hand, concerning the control, this experimental group showed an increase of cDC CD103^+^CD11b^−^ MHC-II cells at weeks 12 and 16 (Figure 4C). However, from weeks 12 to 16, this phenotype of DCs decreased in the SLE group (Figure 4C). As for pDC MHC-II, these cells showed a variable behavior in their number throughout the weeks, reaching their peak at week 12 in both experimental groups (Figure 4D).

Nevertheless, in weeks 12 and 16, the SLE group increased their number (Figure 4D). Referring to MDSCs MHC-II, both phenotypes presented the same behavior in the different weeks in both experimental groups (Figure 4F,G). However, G-MDSC MHC-II acquired an increase from week 10 to week 16 in the SLE group (Figure 4F).

To further understand how these populations are distributed in the spleen, we analyzed the frequency of these populations in this tissue. At week 10, we observed that cDC CD103^−^CD11b^+^ MHC-II^+^ cells represent the highest percentage (Figure 4H) in both groups. However, in the control group, we can see that cDC CD103^+^CD11b^−^ MHC-II^+^ cells presented a very similar percentage to the previously mentioned cells, acquiring the highest percentage impact between both phenotypes (Figure 4H). Meanwhile, the SLE group has low frequencies among the other phenotypes of DCs and MDSCs expressing MHC-II (Figure 4H). Concerning week 12, cDC CD103^−^CD11b^+^ MHC-II^+^ cells again represented the highest percentage of those analyzed in the two experimental groups (Figure 4I). However, pDC MHC-II and moDC MHC-II acquired a higher representation than other cell populations (Figure 4I). Finally, at week 16, as in the other two weeks in both groups, cDC CD103^−^CD11b^+^ MHC-II cells acquired the highest abundance, representing virtually the whole of the cellular spectrum analyzed (Figure 4J).

In axillary lymph nodes, we observed that cDC CD103^−^CD11b^+^ MHC-II^+^ and moDC MHC-II increased in number at week 16 in the SLE group (Figures S2A and S2D, respectively). Meanwhile, cDC CD103^+^CD11b^−^ MHC-II^+^ increased at weeks 10 and 16 (Figure S2B). On the other hand, pDC MHC-II cells, corresponding to the SLE group, decreased throughout the weeks (Figure S2C). However, in weeks 10 and 12, this phenotype of DCs increased in number in the control group (Figure S2C). Regarding MDSCs, the G-MDSC MHC-II phenotype did not show variations at different time points or between experimental groups (Figure S2E). Nevertheless, the number of M-MDSC MHC-II cells is higher at all points evaluated in the SLE group compared to the control (Figure S2F). Additionally, we analyzed the frequency of these cells. At week 10, as in the spleen, cDC CD103^−^CD11b^+^ MHC-II^+^ and cDC CD103^+^CD11b^−^ MHC-II^+^ cells represented the two largest majorities in both experimental groups (Figure S2G). Moreover, in week 12, in the control group, G-MDSC MHC-II cells acquired a higher representation than the other phenotypes (Figure S2H). Meanwhile, in the SLE group, cDC CD103^−^CD11b^+^ MHC-II^+^ and moDC MHC-II cells acquired the highest representation (Figure S2H). During week 16, in the control group, G-MDSC MHC-II cells still represented the highest percentage of cells (Figure S2I). On the other hand, in the SLE group, cDC CD103^−^CD11b^+^ MHC-II^+^ cells continued to display the highest frequency (Figure S2I). These results suggest that the increased number of DCs and MDSCs expressing MHC-II by the SLE group could potentially be impacting pathological development through antigen presentation, highlighting mainly the presence of cDC CD103^−^CD11b^+^ MHC-II^+^ cells, which predominated in frequency in both lymphoid organs.

### 2.4. ICOSL Expression Increases in the Spleen of the MRL/MpJ-Fas^lpr^/J Model During Week 16

To further understand the immunological synapse that could potentially be associated with the populations of DCs and MDSCs that express MHC-II, we analyzed the expression of the genes *ICOSL*, *CD80*, and *CD86*, which encode for activating co-stimulatory molecules relevant to pathology. We observed an increase in the relative expression of *ICOSL* by the SLE group at week 16 of the study (Figure S3A). Meanwhile, the relative expression of *CD80* (Figure S3B) and *CD86* (Figure S3C) were similar throughout the study in both experimental groups. These results suggest that in the MRL/MpJ-*Fas^lpr^*/J murine model, the expression of *ICOSL* might have a predominant role in the lymphocyte activation process during the late stages of the disease compared to *CD80* and *CD86*.

### 2.5. M-MDSC Expressing MHC-II Correlate Positively with IFN-γ-Producing CD4^+^ T Cells in the Spleen of the MRL/MpJ-Fas^lpr^/J Model During Week 16

**Figure 5:**
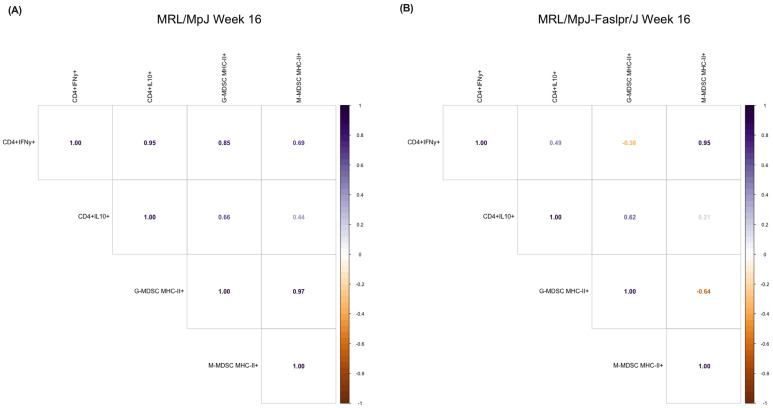
M-MDSC MHC-II positively correlated with IFN-γ-producing CD4^+^ T cells at week 16 from the spleen of the MRL/MpJ-Fas^lpr^/J model. Based on the total number of CD4^+^ T cells and MDSCs expressing MHC-II obtained from the spleen, a correlation was performed at week 16. This analysis assessed the correlation between MDSCs expressing MHC-II concerning IFN-γ- and IL-10-producing CD4^+^ T cells in the (**A**) MRL/MpJ and (**B**) MRL/MpJ-Fas^lpr^/J groups. The values indicate the trend between the cell populations considered. Purple indicates a positive correlation, while orange indicates a negative correlation. MRL/MpJ (*n* = 3), MRL/MpJ-Fas^lpr^/J (*n* = 3). The statistical analysis used was a Pearson correlation.

Based on the increase in *ICOSL* expression during week 16, we sought to understand further the potential relevance of MDSCs expressing MHC-II to T-cell activation during this point. For this purpose, we evaluated the correlation between these cells concerning IFN-γ and IL-10-producing CD4^+^ T cells. We observed that during week 16, the control group presented a positive correlation between G-MDSC MHC-II with both IFN-γ and IL-10-producing CD4^+^ T cells (Figure 5A). On the other hand, the SLE model showed a positive correlation between M-MDSC MHC-II with IFN-γ-producing CD4^+^ T cells (Figure 5B). At the different points of the study, specifically at week 10, the control group showed a behavior similar to that observed at week 16 (Figure S4A). Meanwhile, the SLE model presented negative correlations between MDSCs expressing MHC-II with CD4^+^ T cells (Figure S4B). This same effect was observed in week 12 in both experimental models (Figure S4C,D). These results suggest that in the late stages of SLE, MDSCs expressing MHC-II may promote pathology by activating IFN-γ-producing CD4^+^ T cells.

## 3. Discussion

SLE is an autoimmune disease that can potentially affect different body tissues and does not exclude men or women [1,2]. In this pathology, APCs are hyperactivated, promoting the activation of CD4^+^ T cells via MHC-II, which will induce subsequent tissue damage [7,8,13]. Therefore, it is important to know how different populations of APCs are distributed in secondary lymphoid organs together with the expression of co-stimulatory molecules that could promote the immunological synapse.

Our study utilizing the MRL/MpJ-*Fas^lpr^*/J model showed increased SLE clinical score and proteinuria since week 14 (Figure 1A and Figure 1B, respectively). In particular, increased proteinuria indicates the presence of severe renal damage [27]. This is consistent with the literature since both the MRL/MpJ-*Fas^lpr^*/J model and other murine models of SLE present alterations that induce damage to this tissue [28,29,30,31]. Moreover, this is directly related to what is observed in SLE patients because, in these individuals, the kidney is a severely affected organ [1]. On the other hand, ANA concertation was similar between both experimental groups in the different weeks of the study (Figure 1C). This is not an isolated event, as these self-antibodies have been observed in healthy patients without developing autoimmune disease [32,33]. Additionally, ANA concentration oscillates between weeks, decreasing in week 16 (Figure 1C). This effect has been observed in SLE patients, who present a decrease in ANA titers in the late stages of the pathology [34]. Nevertheless, our SLE model’s anti-dsDNA antibody concentration increases (Figure 1D). Remarkably, the presence of this autoantibody is a relevant criterion for diagnosing SLE since it is specific to the pathology [35,36]. Considering this background and our results, we suggest that our murine model is appropriate for the study of SLE.

For the different cell populations evaluated in our study, UMAP analysis showed that specific populations of DCs in the spleen share some sections of the clusters, especially between cDC CD103^−^CD11b^+^ cells and moDCs (Figure 3A). Both cell populations that may cause this are characterized by CD11b, CD11c, and MHC-II surface markers [37]; especially in this assay, we used CD11b and CD11c for the dimensional reduction analyses. Moreover, we observed that the SLE group has an increase in the different phenotypes of DCs analyzed at different weeks in both axillary lymph nodes (Figure 2A–D) and spleen (Figure 3B–E). These results are opposite to what has been observed in blood from SLE patients since they present a decrease in the frequency of cDCs, pDCs, and moDCs [38,39,40]. However, the contrast of our study with clinical results can be correlated since, in inflammatory conditions, DCs tend to migrate to secondary lymphoid organs [12]. In the SLE murine model, it has been described that F1 (NZW × NZB) mice (BWF1) show an increase in pDCs in spleen and mesenteric lymph nodes [41], which is consistent with that observed in our SLE model (Figure 2C and Figure 3D). Concerning MDSCs, we observed an increase in M-MDSCs in the axillary lymph nodes (Figure 2F) and spleen (Figure 3G) by the MRL/MpJ-*Fas^lpr^*/J model. This increase in M-MDSCs has also been observed in blood from patients with SLE, which correlates with the severity of the pathology [42]. These observations could also be related to our results since the increase in M-MDSCs occurs at week 16, which is the week which shows an increase in clinical score in the SLE model (Figure 1A).

On the other hand, we observed an increased presence of DCs expressing MHC-II in our murine model with SLE at 16 weeks in both spleen (Figure 4B–E) and axillary lymph nodes (Figure S2A–D). This could be because, at this stage, DCs are recruited more significantly to secondary lymphoid organs to present antigens to CD4^+^ T cells via MHC-II [43,44]. Our results indicate that cDC CD11b^+^CD103^−^ MHC-II has a higher preponderance over the other phenotypes in both spleen (Figure 4B–J) and axillary lymph nodes (Figure S2A–I). Although cDC1 (CD103^+^) is considered the most abundant type of DC in lymphoid organs [45], the predominance of cDC CD11b^+^CD103^−^ MHC-II may be because cDC2, which presents CD11b^+^CD103^−^ markers, induces both CD4^+^ T cell activation and polarization towards Th_2_ and Th_17_ phenotypes [12]. Furthermore, it has been observed in patients with Sjögren’s syndrome that cDC2 increases the proliferation of CD4^+^ T cells which subsequently target tissues [46]. Meanwhile, cDC CD103^+^ activates CD4^+^ T cells but strongly involves cross-presenting antigens to CD8^+^ T cells [12,47]. Furthermore, the IL-31/IL-33 axis could impact this effect since these cytokines are altered in SLE, promoting the Th_2_ cell increase [48,49]. Our SLE model presented a rise in pDC MHC-II in both spleen (Figure 4D) and axillary lymph nodes (Figure S2C), peaking at week 12, when they are positioned as the second most abundant cell population in the spleen (Figure 4I). In EAE, pDC MHC-II is recruited to lymph nodes, interacting with CD4^+^ T cells, promoting a regulatory T cell, which secretes IL-10 [50]. Although this cytokine exhibits anti-inflammatory functions par excellence, it has been reported in murine models of SLE that IL-10 promotes the proliferation and differentiation of autoreactive B cells, contributing to the severity of the pathology [51]. Therefore, pDC MHC-II in the SLE model could promote pathology by generating CD4^+^ regulatory T cell phenotypes. On the other hand, moDC MHC-II showed an increase at week 16 in the spleen (Figure 4E) and axillary lymph nodes (Figure S2D), the presence of which could be promoting SLE progression. In rheumatoid arthritis, these cells are involved in an axis involving Th_17_ cells and plasma cells [52]; therefore, in SLE, these moDC MHC-II could promote the inflammatory stages that promote subsequent tissue damage.

Regarding the expression of co-stimulatory molecules, we observed that during weeks 10 and 12, the relative expression of *ICOSL* was similar in both experimental groups (Figure S3A). This phenomenon has been observed in patients with SLE, who have identical ICOSL mRNA levels in peripheral blood compared to healthy subjects [53]. On the other hand, in week 16, we observed an increase in the relative expression of this co-stimulatory molecule (Figure S3A). The increase could be due to the rise in the presence of APCs since, when analyzing these populations in patients with SLE, it was observed that pDCs and myeloid DCs present an increased relative expression of *ICOSL* [54]. If the relative expression of *ICOSL* were proportional to protein expression, this co-stimulatory molecule would be relevant to the development and progression of SLE in our murine model since ICOSL promotes both CD4^+^ T cell activation and proliferation, along with induction of humoral immunity [9,55]. Nonetheless, *CD80* and *CD86* expression was similar throughout the study in both experimental groups (Figure S3B and S3C, respectively). Although CD80 and CD86 are highly expressed molecules in SLE patients [56], in NZM2410 and NZB-W/F1 mice, DCs show a decreased expression of CD80 and a similar expression of CD86 compared to the control [57]. Additionally, it has been observed in the MRL/*lpr* model that the expression of these co-stimulatory molecules decreased in APCs after T-cell stimulation [58]. In this study, control mice of the CBA/J strain were used [58], which could suggest that our control mice of the MRL/MpJ strain could be showing a similar pattern in the expression of CD80 and CD86 for our SLE model. Moreover, it has been observed in chronic conditions, especially in chronic hepatitis B virus (HBV) infection, that modulation of co-stimulatory molecules occurs, resulting in a decrease in CD80 expression [59].

The correlation between the different cellular phenotypes indicated that the control group presented a positive correlation between G-MDSC MHC-II and IFN-γ and IL-10-producing CD4^+^ T cells during week 16 (Figure 5A). This could suggest that this phenotype of MDSCs is more related to a regulatory effect, as IL-10 is a key anti-inflammatory cytokine in inflammatory conditions [60]. Even the IFN-γ producing CD4^+^ T cell phenotype observed in the control group may possess anti-inflammatory properties, because this cytokine has pleiotropic properties in autoimmune diseases [61]. However, this effect was lost in the SLE group (Figure 5B). Although the precise cause of this phenomenon remains unclear, alterations in vitamin D have been suggested as a potential factor. This is supported by observations of decreased vitamin D levels in patients with SLE [62]. Furthermore, vitamin D has been shown to play a protective role against the disease [63,64]. Remarkably, the vitamin D receptor (VDR) is expressed in APCs, promoting a tolerogenic state, particularly in MDSCs [65]. Notably, vitamin D decreases the immunosuppressive activity of MDSCs [66,67], which might contribute to the loss of the protective relationship at week 16 in the spleen of the MRL/MpJ-*Fas^lpr^*/J model.

On the other hand, the SLE group showed a positive correlation between M-MDSC MHC-II with IFN-γ-producing CD4^+^ T cells (Figure 5B). This interaction could promote an inflammatory state, since IFN-γ is known to promote inflammation and is involved in developing autoimmune diseases [68]. In SLE, IFN-γ-secreting Th_1_ cells promote pathology, and this cytokine even inhibits Th_17_ cells [69]. However, it has been proposed that in the long course, IFN-γ-secreting Th_1_ cells promote chronic Th_17_ cell-mediated inflammation [70]. Furthermore, it has been observed that MDSCs, through the ICOSL/ICOS axis, can promote aneurysms by promoting Th_17_ cells [71]. Therefore, the correlation between M-MDSC MHC-II with IFN-γ-producing CD4^+^ T cells may enhance Th_17_ cell activation as pathology progresses.

These results indicate that the MRL/MpJ-*Fas^lpr^*/J murine model shows an accumulation of cDC CD103^−^CD11b^+^ MHC-II^+^ cells, an increase in *ICOSL* expression in the spleen during week 16 of our study, in conjunction with a positive correlation between M-MDSC MHC-II and IFN-γ-producing CD4^+^ T cells, which could be promoting the SLE development.

## 4. Materials and Methods

### 4.1. Mice

We used male and female MRL/MpJ (control) and MRL/MpJ-*Fas^lpr^*/J (SLE) mice, and the strain was obtained from The Jackson Laboratory, Bar Harbor, ME, USA. Animals were maintained under controlled environmental and sterile conditions at a constant temperature of 22 °C, with 12-h light/dark cycles and access to food and water ad libitum in a pathogen-free facility at Pontificia Universidad Católica de Chile. Euthanasia was performed at different points in time (weeks 10, 12, and 16) through an intraperitoneal injection made of ketamine (80 mg/kg) and xylazine (4 mg/kg) mix. The clinical score of SLE monitoring was carried out 3 times weekly from week 8 until week 16. The analyzed parameters included weight loss, appearance and behavior, lymph node size, skin lesions, and proteinuria (Table S1). All procedures were performed following the animal handling manual of the Bioethics Committee of the Universidad Andrés Bello (021/2022) and Pontificia Universidad Católica de Chile (CEC 220728001).

### 4.2. Sample Collection

Cells were obtained from the spleen and axillary lymph nodes. Both tissues were homogenized mechanically using a 70 μm cell strainer. The resulting flowthrough obtained from homogenization was incubated with 700 μL of 1× Ammonium-Chloride-Potassium (ACK) buffer for 5 min at room temperature (RT) to lyse erythrocytes, and the reaction was subsequently stopped by the addition of 700 μL of 1× PEB buffer (0.5% bovine serum albumin, 2 mM EDTA, and PBS 1× at pH 7.4). Cells were centrifuged at 300× *g* at 4 °C for 5 min. Finally, the cells were resuspended in 1× PEB buffer and stored until further use. Meanwhile, to analyze anti-dsDNA and ANA, blood samples were recovered by cardiac puncture, incubated at 37 °C for 30 min, and centrifuged at 2200× *g* for 15 min, aiming to obtain serum.

### 4.3. Measure of Anti-dsDNA and ANA

The concentration of anti-dsDNA antibodies and ANA was determined by ELISA. To analyze anti-dsDNA antibodies, we added 0.1 mg/mL of Calf Thymus DNA antigen (Invitrogen, Carlsbad, CA, USA, Cat #15633-019) to each well of the plate. For the calibration curve, we added a 1:4000 dilution of mouse anti-dsDNA antibody (Abcam, Cambridge, UK, Cat#AB27156). Simultaneously, we used a dilution of 1:500 mice serum samples. Subsequently, we added a 1:2000 dilution of a secondary antibody HRP goat anti-mouse IgG (Invitrogen, Carlsbad, CA, USA, Cat#62-6520) and incubated for 50 min. Later, we added 100 μL of Ultra TMB (Thermo Scientific, Waltham, MA, USA, Cat #34029), and the samples were incubated at RT in the dark for 10 min. Finally, we added 50 μL of stop solution (H_2_SO_4_ 2N). For ANA, we performed a 1:10 dilution with sample diluent (CUSABIO, Houston, TX, USA, Cat#CSB-E12912m), and we quantified these antibodies following the manufacturer’s instructions (CUSABIO, Houston, TX, USA, Cat#CSB-E12912m). Afterward, the plates were analyzed with a spectrophotometer (Epoch, BioTek, Hong Kong, China) at a 450/570 nm wavelength.

### 4.4. Flow Cytometry

Three panels were performed each week: one for DCs, one for MDSCs, and one for T cells (Table 1 and Table 2, and Table 3, respectively). The cells were obtained from the spleen and lymph nodes and were incubated with viability stain (BD Horizon^TM^ Fixable Viability Stain 700, San Jose, CA, USA) at 4 °C for 20 min in darkness. Subsequently, cells were washed with 1× PEB and incubated with antibodies for 1 h at 4 °C to stain the surface antigen. We fixed and permeabilized the cells for intracellular labeling using a Fixation/Permeabilization Kit (BD Cytofix/Cytoperm™, San Jose, CA, USA). Afterward, we added the intracellular antibodies and incubated overnight at 4 °C. Finally, cells were resuspended in PEB 1× in conjunction with CountBright Counting Beads (ThermoFisher, Waltham, MA, USA, Cat# C36950) (dilution 1:30) to normalize the data obtained by the flow cytometer. Data quality control in FlowJo v10.10 was performed by PeacoQC v1.5. FlowSOM v4.1 was used to perform Uniform Manifold Approximation and Projection for dimensional reduction (UMAP) and incorporate the cluster exploration into FlowJo software. The gating strategy for the different cells was as follows: cDC CD11b^+^CD103^−^ (CD45^+^CD11b^+^CD11c^+^CD64^+^CD103^−^/MHC-II^+^), cDC CD11b^−^CD103^+^ (CD45^+^CD11b^−^CD11c^+^CD103^+^/MHC-II^+^), pDCs (CD45^+^CD11b^−^CD11c^+^Ly6C^+^/MHC-II^+^), moDCs (CD45^+^CD11b^+^CD11c^int^Ly6C^+^/MHC-II^+^), G-MDSCs (CD45^+^CD11b^+^Ly6G^+^Ly6C^low^/MHC-II^+^), M-MDSCs (CD45^+^CD11b^+^Ly6G^−^Ly6C^high^/MHC-II^+^), IFN-γ producing CD4^+^ T cells (CD45^+^CD3^+^CD4^+^IFN-γ^+^), and IL-10 producing CD4^+^ T cells (CD45^+^CD3^+^CD4^+^IL-10^+^) (Figure S5).

### 4.5. RNA Extraction

A spleen section was collected and stored at −80 °C in 500 μL of TRIzol (Invitrogen, Carlsbad, CA, USA, Cat #15596018) until subsequent use. Thereafter, the samples were homogenized using a hand-held homogenizer (Benchmark Scientific, Edison, NJ, USA). Afterward, we added 100 μL chloroform and incubated for 3 min at RT. Samples were centrifuged at 12,000× *g* for 15 min at 4 °C. The aqueous phase was recovered, 250 μL of isopropanol was added, homogenized by inversion, and incubated for 4 h at −20 °C. Subsequently, the samples were centrifuged at 12,000× *g* for 10 min at 4 °C. The supernatant was discarded and resuspended in 500 μL 75% ethanol. We centrifuged the samples at 7500× *g* for 5 min at 4 °C. We discarded the supernatant and allowed the pellet to dry for 15 min. Finally, we added 50 μL DNase/RNase-Free Distilled Water (Invitrogen, UltraPure^TM^, Carlsbad, CA, USA, Cat #10977-015).

### 4.6. RT-qPCR

We used iScript^TM^ Supermix (Bio-Rad, Hercules, CA, USA) to obtain the cDNA following the manufacturer’s instructions. Specific primers were used to detect *ICOSL*, *CD80*, and *CD86* (Table 4). The products of these molecules were detected using SSO advanced universal SYBR (Bio-Rad) in a Biosystem StepOnePlus Real-Time PCR system relative by CFX opus 96 (Bio-Rad) and StepOnePlus Real-Time PCR System (Applied Biosystems by ThermoFisher). The abundance of *ICOSL*, *CD80*, and *CD86* mRNAs was determined by relative expression to the respective housekeeping gene β-2 microglobulin [72].

### 4.7. Statistical Analysis

Statistical analyses were performed using Prism 10 software (Graph Pad Software, Inc., San Diego, CA, USA). We used two-way ANOVA and Tukey’s post-test to compare each group for analysis. Values are represented as mean standard error of the mean (SEM) and were considered statistically significant when *p* < 0.05. For correlation analysis, we utilized the Pearson correlation using the R package ‘corrplot’ (Version 0.92) in R studio.

## 5. Conclusions

The MRL/MpJ-*Fas^lpr^*/J murine model determined the presence of diverse phenotypes of DCs and MDSCs expressing MHC-II in secondary lymphoid organs throughout SLE development, within which cDC CD103^−^CD11b^+^ MHC-II^+^ cells were established as the most predominant population in terms of cell number within secondary lymphoid organs throughout the study. Additionally, *ICOSL* presented an increase in relative expression throughout the pathology, which was more predominant in week 16. These findings indicate that antigen presentation via MHC-II is led by cDC CD103^−^CD11b^+^ cells and is favored by *ICOSL*, which may contribute to the development and progression of the pathology. This could promote Th_2_ and Th_17_ cell proliferation and polarization in the pathology, enhancing the inflammatory environment and subsequent tissue damage. Moreover, M-MDSC MHC-II correlated positively with IFN-γ-producing CD4^+^ T cells during week 16 of the study, which may indicate that these cells may promote an inflammatory stage. In the future, it would be interesting to perform similar screening in samples from SLE patients to determine if these data can be extrapolated to humans and to determine the mechanism of action of these cells in these individuals.

## Figures and Tables

**Table 1:** Antibodies used to characterize DCs (spleen and lymph nodes) and MDSCs (lymph nodes).

	Antibody	Fluorochrome	Clone	Source
week 10	CD45	BV510	30-F11	BD Biosciences San Jose, CA, USA
	CD11b	APC-Cy7	M1/70	BD Biosciences
	CD11c	PE-Cy7	HL3	BD Biosciences
	CD64	APC	S18017D	BioLegend San Diego, CA, USA
	CD103	BV421	M290	BD Biosciences
	Ly6C	PE	AL-21	BD Biosciences
	Ly6G	PerCP-Cy5.5	1A8	BD Biosciences
	MHC-II	BV605	2G9	BD Biosciences
week 12 and 16	CD45	BV510	30-F11	BD Biosciences
	CD11b	APC-Cy7	M1/70	BD Biosciences
	CD11c	PE-Cy7	HL3	BD Biosciences
	CD64	APC	S18017D	BioLegend
	CD103	BV421	M290	BD Biosciences
	Ly6C	BV605	AL-21	BD Biosciences
	Ly6G	BV786	1A8	BD Biosciences
	MHC-II	PerCP-Cy5.5	M5/144.15.2	BD Biosciences

**Table 2:** Antibodies used for MDSCs in the spleen.

	Antibody	Fluorochrome	Clone	Source
10, 12 and 16 weeks	CD45	BV510	30-F11	BD Biosciences
	CD11b	FITC	M1/70	BD Biosciences
	Ly6C	BV605	AL-21	BD Biosciences
	Ly6G	BV786	1A8	BD Biosciences
	Arg-1	PE-Cy7	A1exF5	Invitrogen
	iNOS	APC	CXNFT	Invitrogen
	MHC-II	PerCP-Cy5.5	M5/144.15.2	BD Biosciences

**Table 3:** Antibodies used for T cells in the spleen.

	Antibody	Fluorochrome	Clone	Source
10, 12 and 16 weeks	CD45	BV510	30-F11	BD Biosciences
	CD3	BV711	145-2C11	BD Biosciences
	CD4	BV650	RM4-5	BD Biosciences
	IFN-γ	PE-CF594	XMG1.2	BD Biosciences
	IL-10	PE	JES5-16E3	BioLegend

**Table 4:** Primers used for the genes of interest.

Gene	Forward Primer	Reverse Primer
*ICOSL*	TCTTGGAAGAGGTGGTCAGGCT	GCCATTCTTGGAGGACATGCAGGT
*CD80*	TGCTGCTTCGTCTTTCAC	GAGGAGAGTTGTAACGGCAAG
*CD86*	AAAGTTGGTTCTGTACGAGCA	GGCCCAGGTACTTGGCATT
*β-2 microglobulin*	CATGGCTCGCTCGGTGAC	CAGTTCAGTATGTTCGGCTTCC

## Data Availability

The data presented in this study are available upon request from the corresponding author.

## Supplementary Materials

The supporting information can be downloaded at https://www.mdpi.com/article/10.3390/ijms252413604/s1.
