# Supplementary material for: Characterization of Dendritic Cells and Myeloid-Derived Suppressor Cells Expressing Major Histocompatibility Complex Class II in Secondary Lymphoid Organs in Systemic Lupus Erythematosus-Prone Mice
Source: Int J Mol Sci. 2024 Dec 19;25(24):13604. doi: 10.3390/ijms252413604 (PMC11676837; doi:10.3390/ijms252413604)
Supplement: Supplementary file 1 [file ijms-25-13604-s001.zip › Supplementary Material Uribe, F, et al VF.pdf]

Supplementary Figures

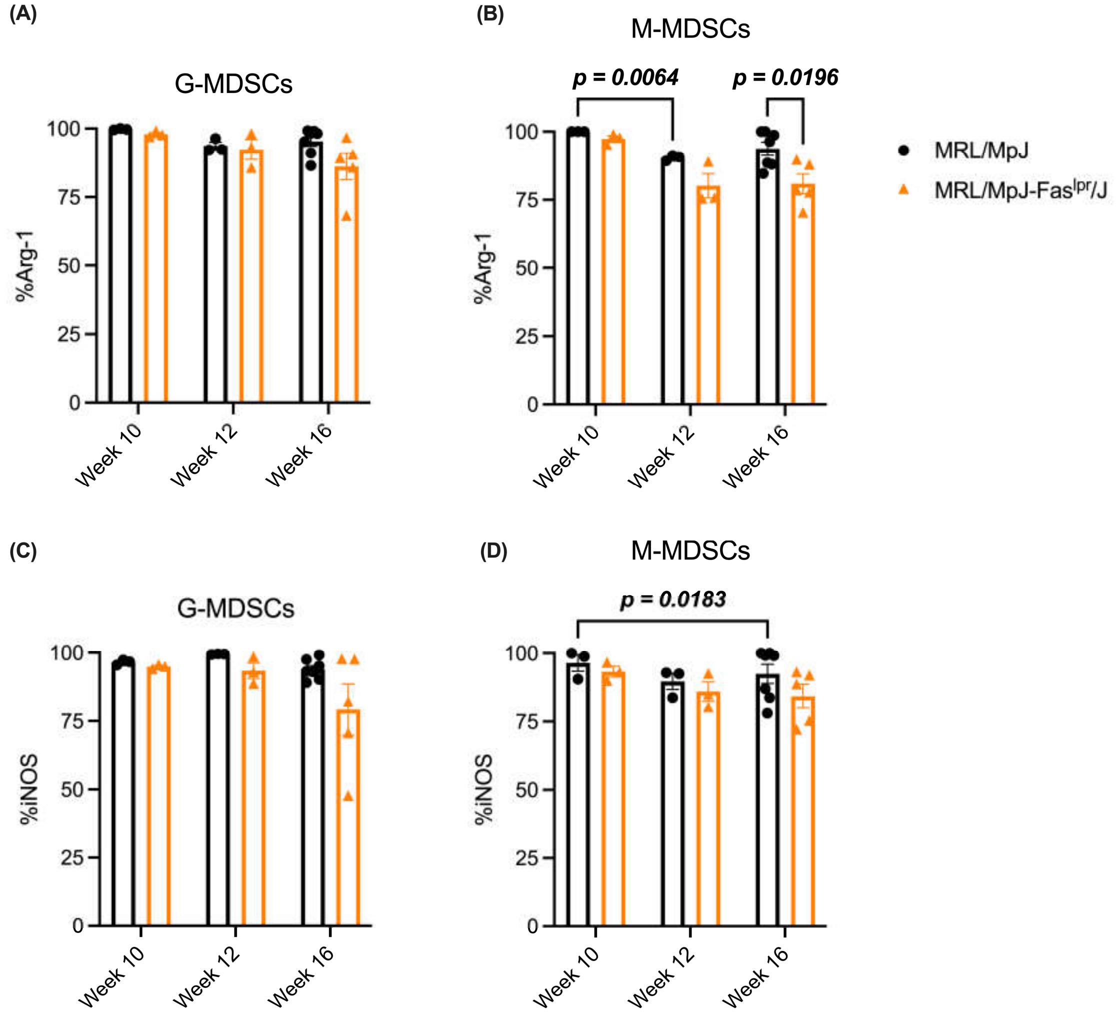

Figure S1. The selected G-MDSCs in both experimental groups presented Arg-1 and iNOS markers in their totality. The MDSC, Arg-1, and iNOS immunosuppression markers were analyzed from the chosen MDSCs. Percentage of **(A)** G-MDSCs and **(B)** M-MDSCs expressing Arg-1, respectively. Percentage of **(C)** G-MDSCs and **(D)** M-MDSCs expressing iNOS, respectively. MRL/MpJ (black circles) and MRL/MpJ-*Fas^lpr*/J (orange triangles). MRL/MpJ week 10 (n=3), MRL/MpJ-*Fas^lpr*/J week 10 (n=3), MRL/MpJ week 12 (n=3), MRL/MpJ-*Fas^lpr*/J week 12 (n=3), MRL/MpJ week 16 (n=7), MRL/MpJ-*Fas^lpr*/J week 16 (n=5). The statistical analysis performed was two-way ANOVA, post-Tukey's test, p<0.05 (bold).

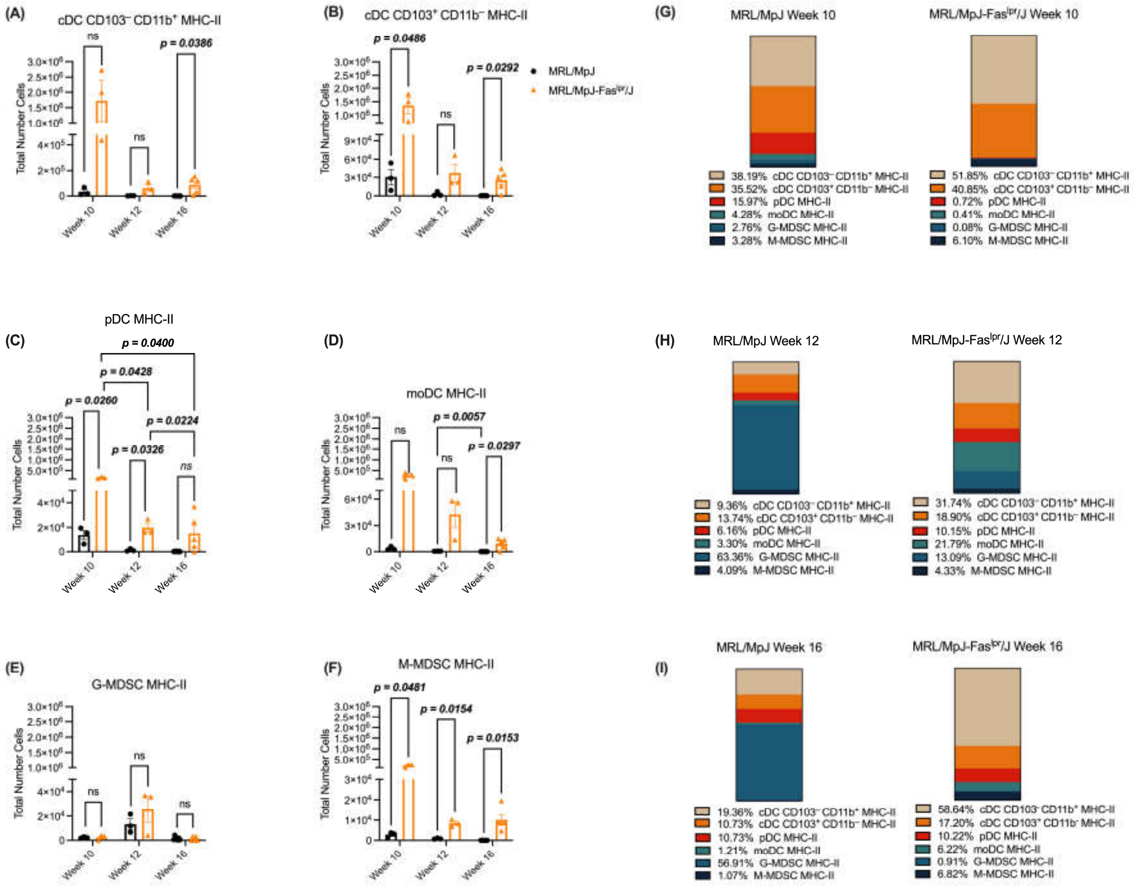

Figure S2. The cDC CD103⁻CD11b⁺ MHC-II cells represented the most abundant population in the axillary lymph nodes of the MRL/MpJ-*Fas^lpr*/J model. The abundance of DCs and MDSCs expressing MHC-II throughout the pathology was analyzed from the axillary lymph nodes of both murine models. A total number of **(A)** cDC $CD103^-CD11b^+MHC\text{-}II^+$, **(B)** cDC $CD103^+CD11b^-MHC\text{-}II^+$, **(C)** pDC MHC-II, **(D)** moDC MHC-II, **(E)** G-MDSC MHC-II, and **(F)** M-MDSC MHC-II cells, respectively. Data are presented across weeks 10, 12, and 16. MRL/MpJ group (black circles) and the MRL/MpJ-*Fas^lpr*/J group (orange triangles). MRL/MpJ week 10 (n=3), MRL/MpJ-*Fas^lpr*/J week 10 (n=3), MRL/MpJ week 12 n=3, MRL/MpJ-*Fas^lpr*/J week 12 (n=3), MRL/MpJ week 16 (n=7), MRL/MpJ-*Fas^lpr*/J week 16 (n=5). The statistical analysis used was a two-way ANOVA, post Tukey's test. $p<0.05$ (bold), "ns" indicated not significant. **(G)-(I)** Frequency of analyzed cell populations expressing MHC-II in both experimental models at weeks 10, 12, and 16, respectively.

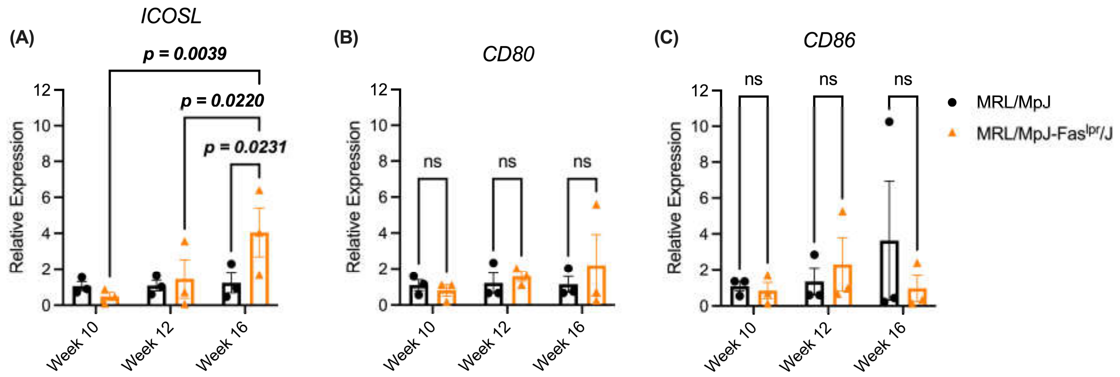

Figure S3. *ICOSL* shows an increase throughout the weeks in the spleen of the SLE model. The spleen's relative expression of different co-stimulatory molecules was determined throughout the SLE**.** Relative expression concerning β-2 microglobulin for **(A)** *ICOSL*, **(B)** *CD80*, and **(C)** *CD86* genes at different points in the study. MRL/MpJ (black circles) and MRL/MpJ-Fas^{lpr}/J (orange triangles). MRL/MpJ week 10 (n=3), MRL/MpJ-*Fas*^{lpr}/J week 10 (n=3), MRL/MpJ week 12 (n=3), MRL/MpJ-*Fas*^{lpr}/J week 12 n=3, MRL/MpJ week 16 (n=3), MRL/MpJ-*Fas*^{lpr}/J week 16 (n=3). The statistical analysis used was a two-way ANOVA post-Tukey's test, $p<0.05$ (bold), "ns" indicated not significant.

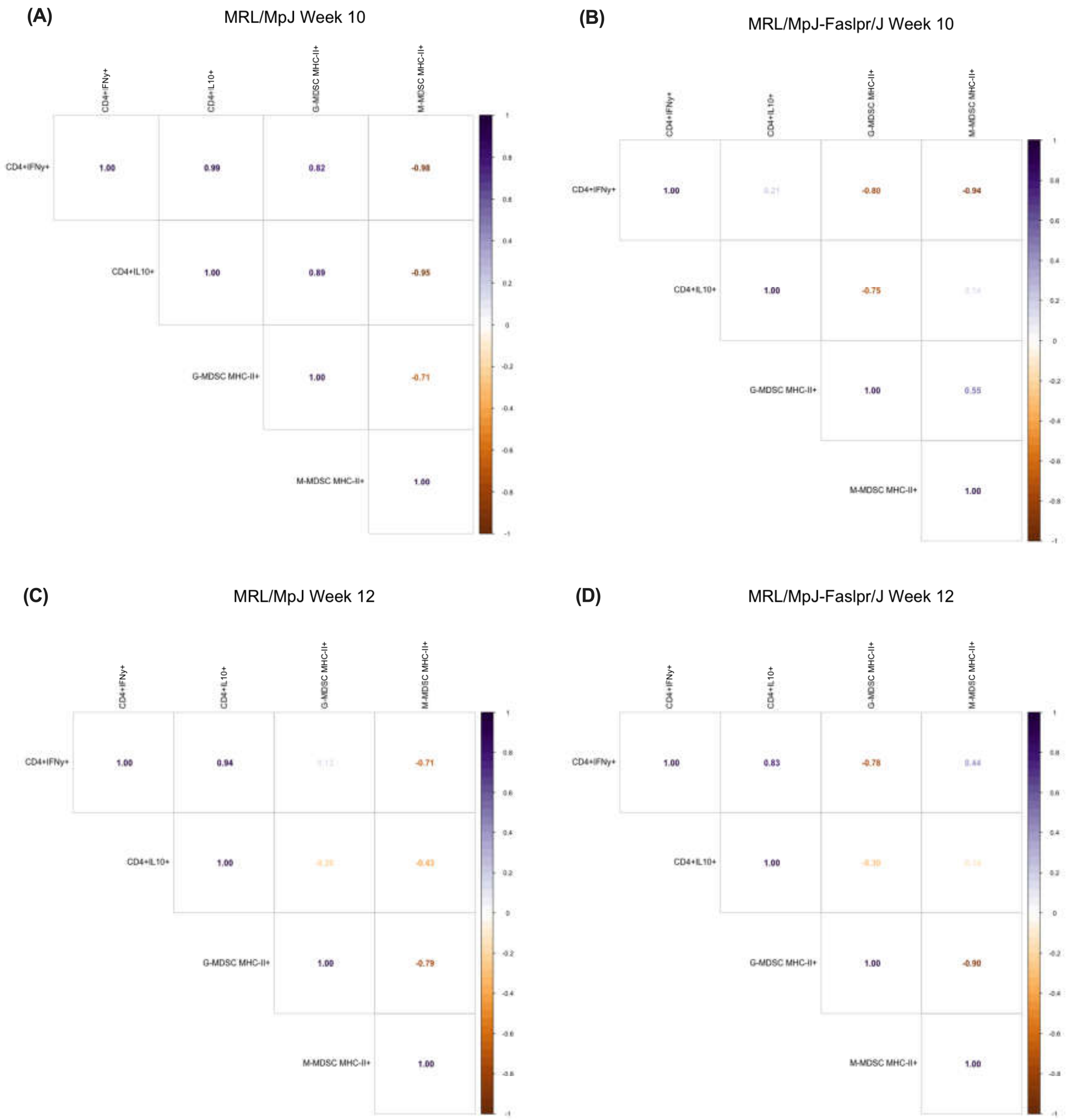

Figure S4. MDSCs expressing MHC-II do not correlate positively with $CD4^{+}$ T cells at weeks 10 and 12 of the MRL/MpJ-*Fas^{lpr}*/J mouse model study. Based on the total number of $CD4^{+}$ T cells and MDSCs expressing MHC-II obtained from the spleen, a correlation was performed at weeks 10 and 12. This analysis assessed the correlation between MDSCs expressing MHC-II concerning IFN-γ- and IL-10-producing $CD4^{+}$ T cells in the (A) MRL/MpJ week 10, (B) MRL/MpJ-*Fas^{lpr}*/J week 10 (C) MRL/MpJ week 12 and (D) MRL/MpJ-*Fas^{lpr}*/J week 12 groups. The values indicate the trend between the confronted cell populations. Purple color indicates a positive correlation, while orange indicates a negative correlation. MRL/MpJ week 10 (n=3), MRL/MpJ-*Fas^{lpr}*/J week 10 (n=3), MRL/MpJ week 12 (n=3), and MRL/MpJ-*Fas^{lpr}*/J week 12 (n=3) . The statistical analysis used was a Pearson correlation.

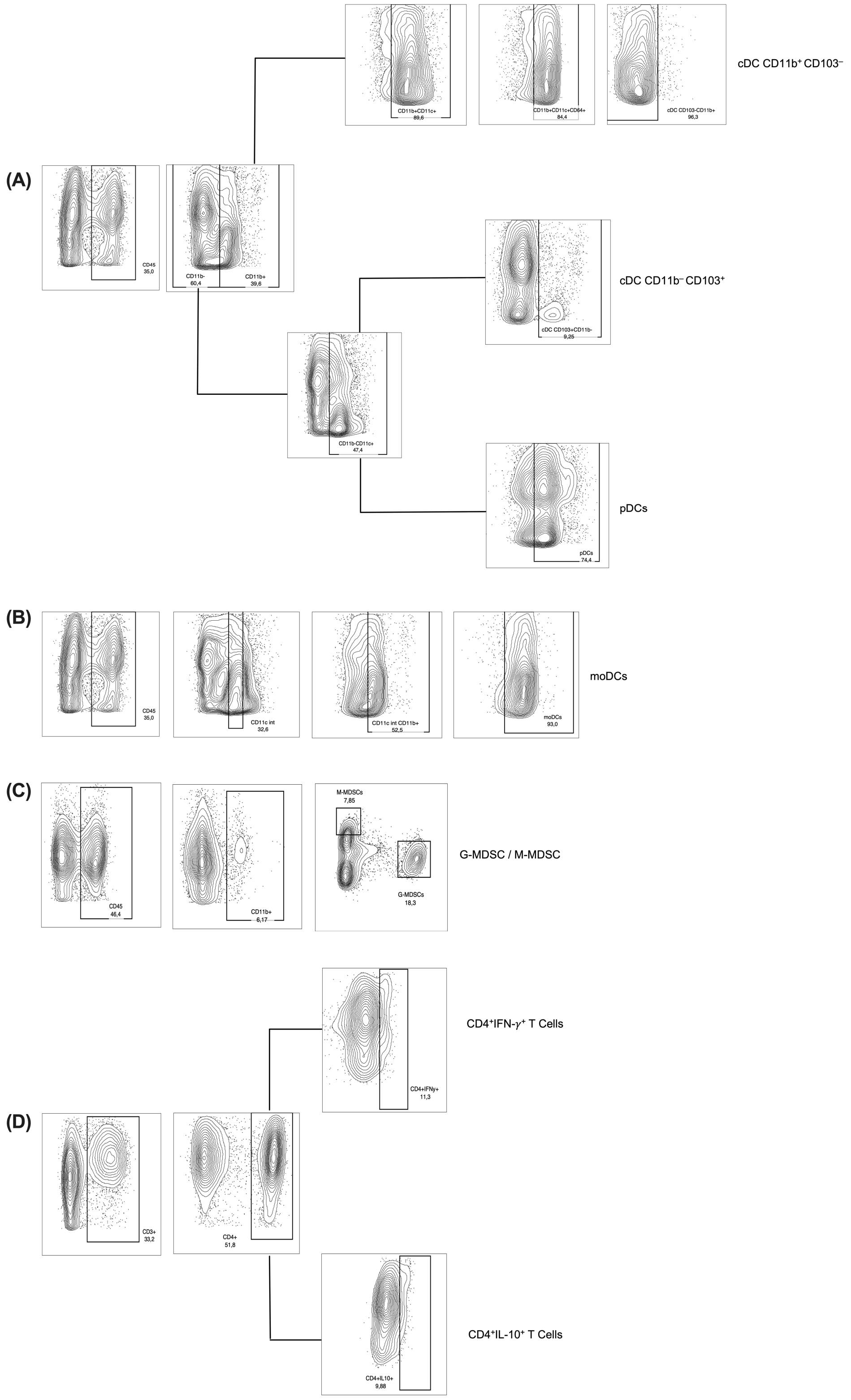
(A)
CD45
35,0
CD11b-
60,4
CD11b+
39,6
CD11b+CD11c+
89,6
CD11b+CD11c+CD64+
84,4
cDC CD103-CD11b+
96,3
cDC CD11b⁺ CD103⁻
CD11b-CD11c+
47,4
cDC CD103+CD11b-
9,25
cDC CD11b⁻ CD103⁺
pDCs
74,4
pDCs
(B)
CD45
35,0
CD11c int
32,6
CD11c int CD11b+
52,5
moDCs
93,0
moDCs
(C)
CD45
46,4
CD11b+
6,17
M-MDSCs
7,85
G-MDSCs
18,3
G-MDSC / M-MDSC
(D)
CD3+
33,2
CD4+
51,8
CD4+IFNγ+
11,3
CD4⁺IFN-γ⁺ T Cells
CD4+IL10+
9,88
CD4⁺IL-10⁺ T Cells

Figure S5. Gating strategy for DCs, MDSCs, and T cells. We utilized flow cytometry to perform the gating strategy of the different DCs, MDSCs, and T cell populations in both experimental models. The images represent the spleen's selections corresponding to the MRL/MpJ-Fas^{lpr}/J model. **(A)** The different phenotypes of DCs were selected from the $CD45^{+}$ marker. $CD11b^{+}CD103^{-}$ cDC populations were obtained considering $CD11b^{+}$ $CD11c^{+}CD64^{+}$ and $CD103^{-}$ markers. On the other hand, $CD11b^{-}CD103^{+}$ cDC cells were selected using $CD11b^{-}$ $CD11c^{+}CD103^{+}$ antibodies. Meanwhile, $CD11b^{-}CD11c^{+}$Ly6C were considered pDCs. (**B**) moDCs were selected from the $CD45^{+}$ population, then CD11c ^{int} (intermediate) markers were selected along with $CD11b^{+}$ and $Ly6C^{+}$ cells. **(C)** MDSCs were selected from the $CD45^{+}CD11b^{+}$ populations; for the granulocytic profile (G-MDSCs), the $Ly6C^{low}$ and $Ly6G^{+}$ antibodies were used. For the monocytic phenotype (M-MDSCs), $Ly6C^{high}$ and $Ly6C^{-}$ were selected. **(D)** $CD4^{+}$ T cell phenotypes were selected from $CD45^{+}CD3^{+}CD4^{+}$ cells; subsequently, we used an IFN-γ or IL-10 antibody, respectively.

Supplementary Table

Table S1. Parameters of clinical score

Parameter	Observation	Score
**Weight Loss**	Normal weight.	0
	2% to 10% weight loss.	1
	11% to 15% weight loss.	2
	16% to 20% weight loss.	3
**Appearance and Behavior**	Normal.	0
	Unkempt coat, shallow breathing, a slight reduction in mobility, and slight behavioral changes.	1
	Hirsute coat, rapid abdominal breathing, mild dehydration, restlessness.	2
	Abnormal posture, labored and irregular breathing, severe dehydration, lethargy, and vocalizations.	3
**Lymph Nodes (cervical, axillary and inguinal)**	Normal.	0
	Small papillary lymph nodes < 1cm in two or more sites.	1
	Big lymph nodes > 1cm in one or more bilateral sites with small lymph nodes.	2
	Big lymph nodes in two or more bilateral sites together.	3
**Skin Lesions**	Normal.	0
	One or two lesions with a total area less than or equal to 0.5 cm, without skin ulceration.	1
	0.5 to 1 cm lesions with skin compromise.	2
	> 1 cm lesions with skin ulceration.	3
**Proteinuria**	Negative	0
	Positive (+)	1
	Positive (++)	2
	Positive (+++)	3
