# Supplementary figures and images for: Characterization of Dendritic Cells and Myeloid-Derived Suppressor Cells Expressing Major Histocompatibility Complex Class II in Secondary Lymphoid Organs in Systemic Lupus Erythematosus-Prone Mice
Source: Int J Mol Sci. 2024 Dec 19;25(24):13604. doi: 10.3390/ijms252413604 (PMC11676837; doi:10.3390/ijms252413604)

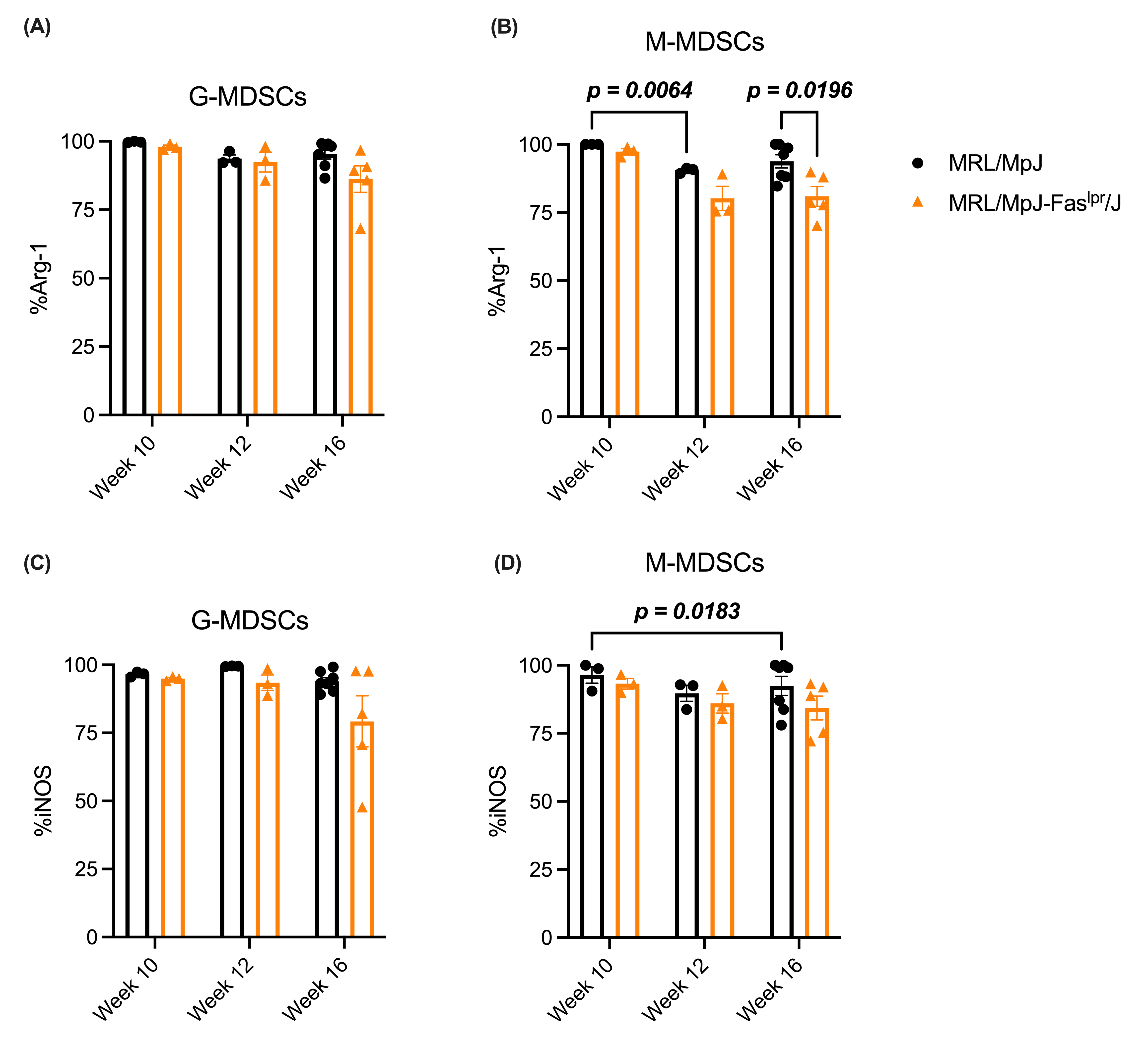

Supplement: Supplementary file 1 [file ijms-25-13604-s001.zip › Figure S1.tiff]

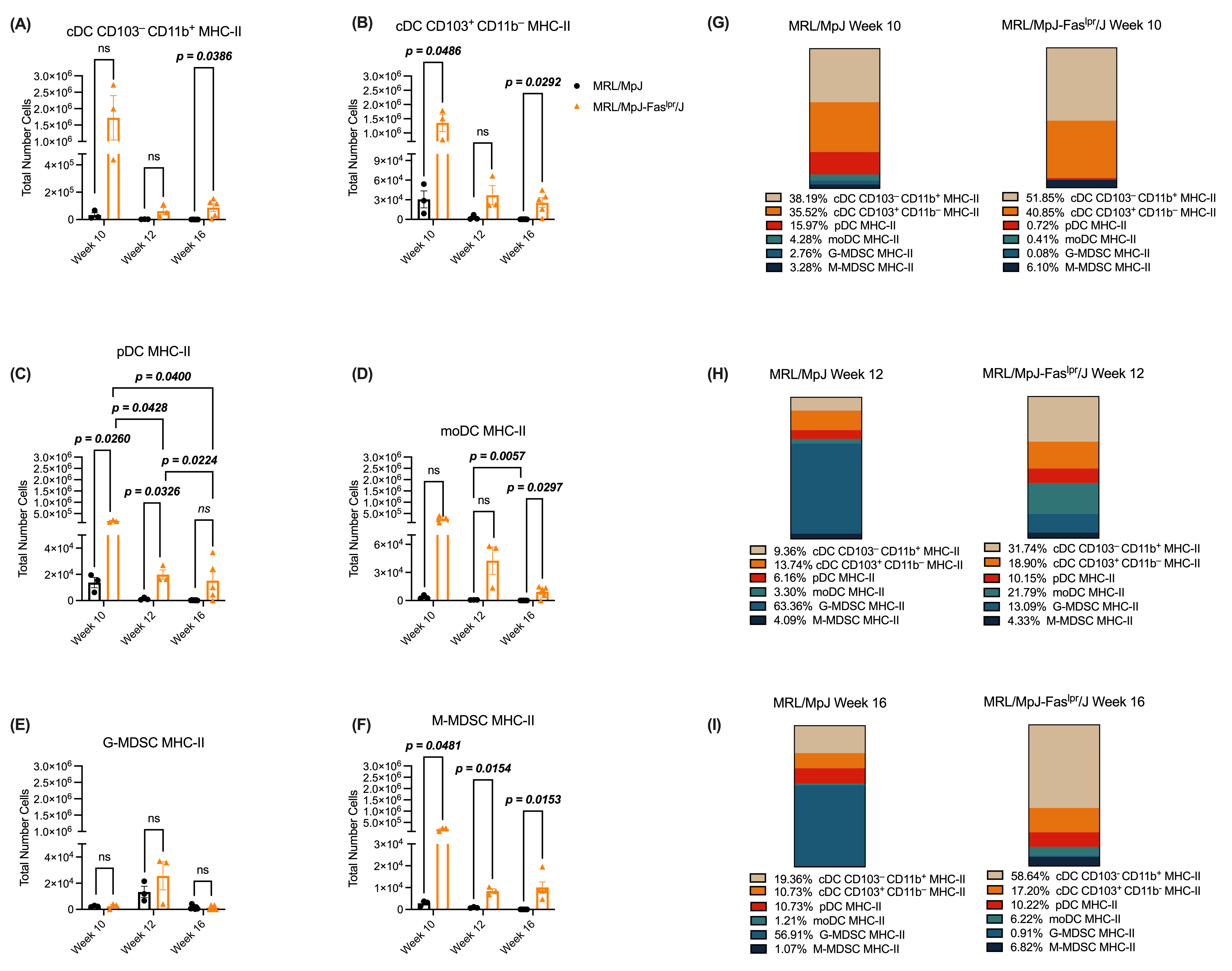

Supplement: Supplementary file 1 [file ijms-25-13604-s001.zip › Figure S2.tiff]

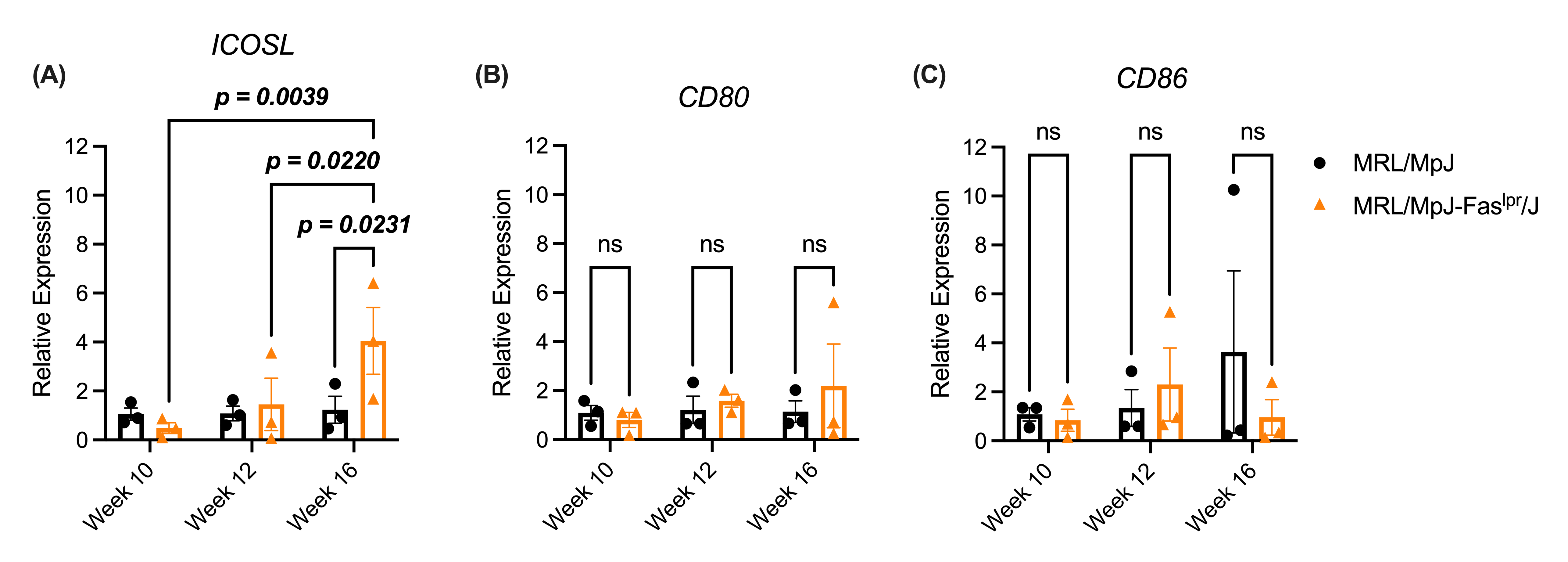

Supplement: Supplementary file 1 [file ijms-25-13604-s001.zip › Figure S3.tiff]

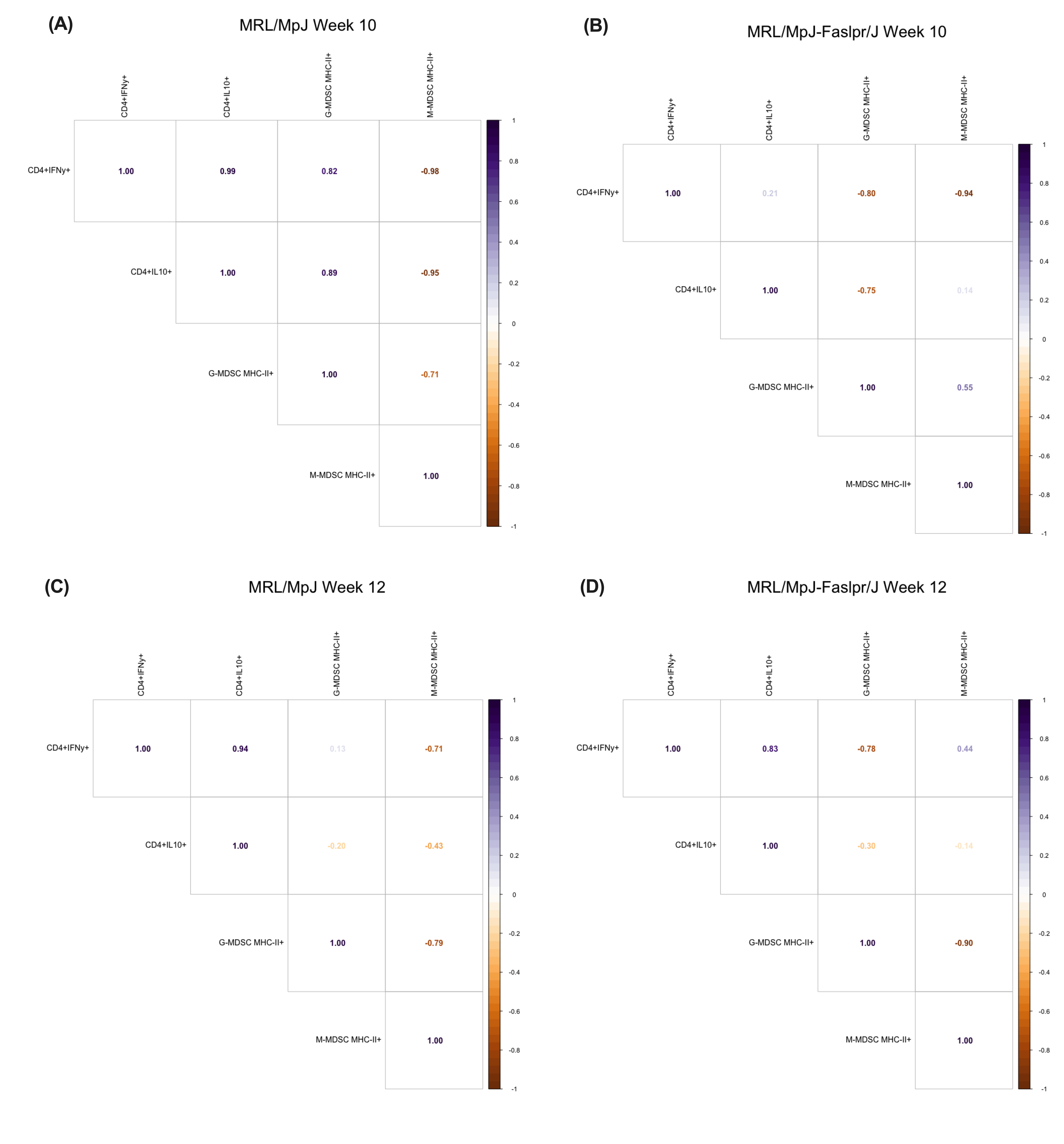

Supplement: Supplementary file 1 [file ijms-25-13604-s001.zip › Figure S4.tiff]

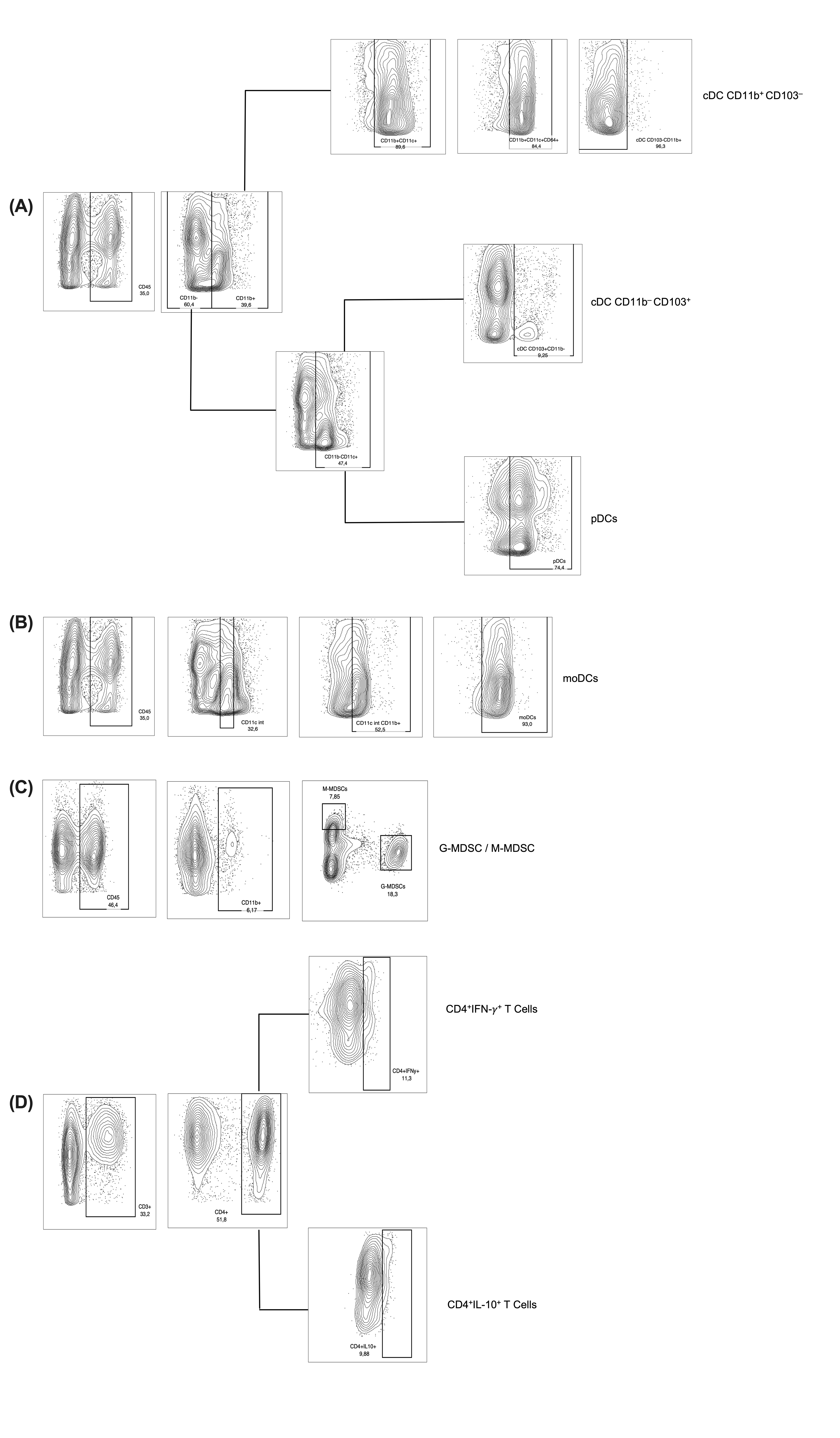

Supplement: Supplementary file 1 [file ijms-25-13604-s001.zip › Figure S5.tiff]
